# Diverse geometric changes related to dynamic left ventricular outflow tract obstruction without overt hypertrophic cardiomyopathy

**DOI:** 10.1186/1476-7120-12-23

**Published:** 2014-07-03

**Authors:** Jung-Joon Cha, Hyemoon Chung, Young Won Yoon, Ji Hyun Yoon, Jong-Youn Kim, Pil-Ki Min, Byoung-Kwon Lee, Bum-Kee Hong, Se-Joong Rim, Hyuck Moon Kwon, Eui-Young Choi

**Affiliations:** 1 Division of Cardiology, Heart Center, Gangnam Severance Hospital, Yonsei University College of Medicine, Seoul, Republic of Korea

**Keywords:** Dynamic left ventricular outflow tract obstruction, Sigmoid septum, Aging

## Abstract

**Background:**

Dynamic left ventricular (LV) outflow tract (LVOT) obstruction (DLVOTO) is not infrequently observed in older individuals without overt hypertrophic cardiomyopathy (HCM). We sought to investigate associated geometric changes and then evaluate their clinical characteristics.

**Methods:**

A total of 168 patients with DLVOTO, which was defined as a trans-LVOT peak pressure gradient (PG) higher than 30 mmHg at rest or provoked by Valsalva maneuver (latent LVOTO) without fixed stenosis, were studied. Patients with classical HCM, acute myocardial infarction, stress induced cardiomyopathy or unstable hemodynamics which potentially induce transient-DLVOTO were excluded.

**Results:**

Their mean age was 71 ± 11 years and 98 (58%) patients were women. Patients were classified as pure sigmoid septum (n = 14) if they have basal septal bulging but diastolic thickness less than 15 mm, sigmoid septum with basal septal hypertrophy for a thickness ≥15 mm (n = 85), prominent papillary muscle (PM) (n = 20) defined by visually large PMs which occluded the LV cavity during systole or 1/2 LVESD, or as having a small LV cavity with concentric remodelling or hypertrophy (n = 49). The prominent PM group was younger, had a higher S’ and lower E/e’ than other groups. In all groups, a higher peak trans-LVOT PG was related (p < 0.10) to higher E/e’, systolic blood pressure, relative wall thickness, and pulmonary arterial systolic pressure. In multivariate analysis, resting trans-LVOT PG correlated to pulmonary arterial pressure (ß = 0.226, p = 0.019) after adjustment for systolic blood pressure, relative wall thickness, and E/e’.

**Conclusions:**

DLVOTO develops from various reasons, and patients with prominent PMs have distinct characteristics. We suggest to use DLVOTO-relieving medication might reduce pulmonary pressure in this group of patients.

## Background

Dynamic left ventricular (LV) outflow tract (LVOT) obstruction (DLVOTO) is not uncommonly observed in aged individuals without overt hypertrophic cardiomyopathy (HCM) [1]. Any conditions which reduce LV cavity size in combination with hypercontractility can induce resting D LVOTO, or it can be provoked by preload manipulation or inotropic stimulation in what is termed latent DLVOTO [1-4]. Aging is a well-matched condition to develop DLVOTO in terms of concentric remodelling and basal septal bulging due to an increased angle to the ascending aorta [5,6]. These echocardiographic cases were frequently described simply as having a sigmoid septum or basal septal hypertrophy without strict criteria. However, even in this setting of ambiguous clinical categorization, there are clearly diverse morphological subtypes of outflow tract obstruction resulting from diverse etiologies. The geometrical determinants and clinical implications have not been fully investigated. Therefore, in this study, we sought to investigate associated geometric changes and classify DLVOTO subgroups in detail and then evaluate their clinical characteristics.

## Methods

**Figure 1:**
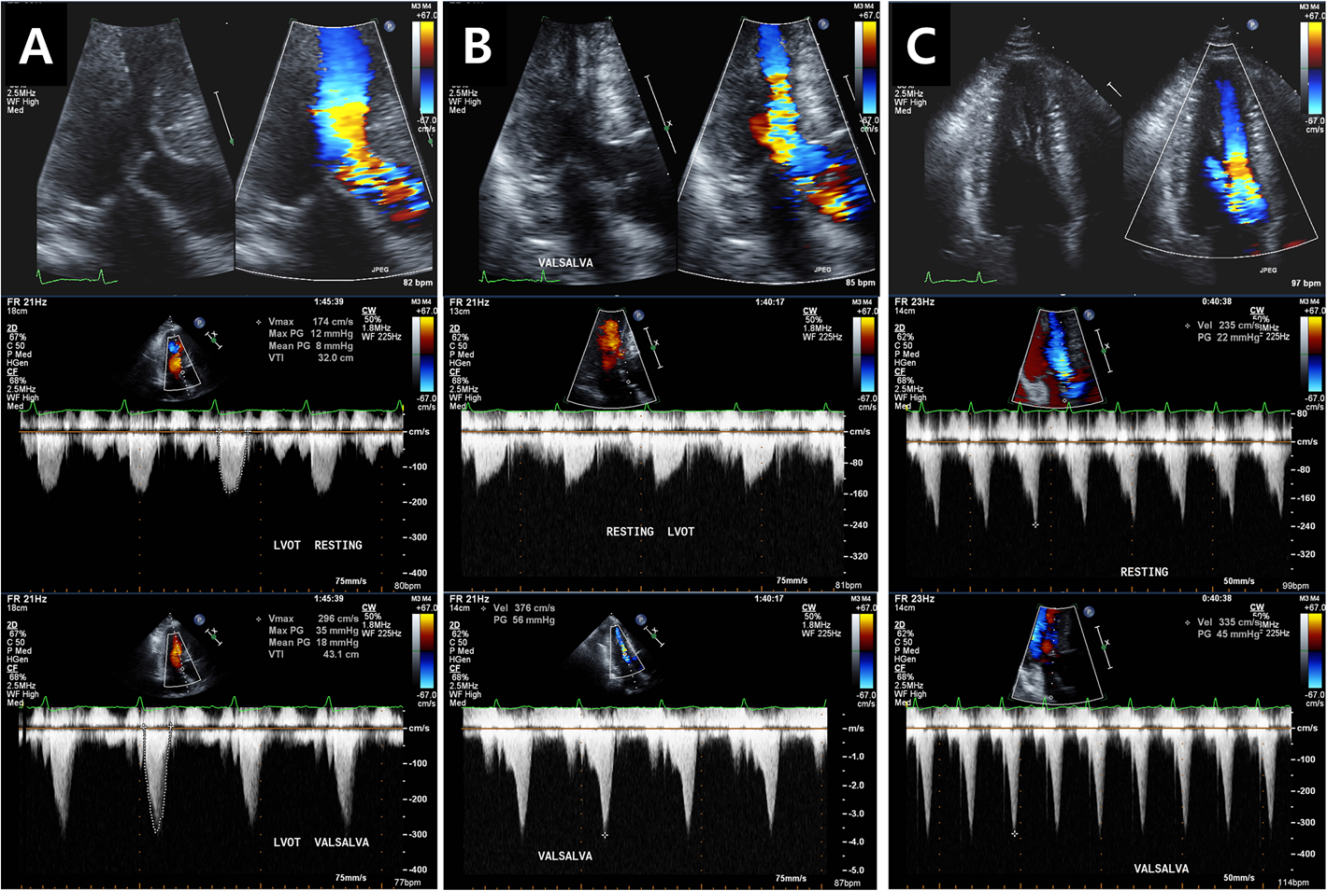
**Representative cases of DLVOTO with Doppler patterns at rest and during Valsalva maneuver.** A case with leaflet systolic anterior motion (SAM) **(A)**, a case with chordal SAM **(B)**, and a case with prominent papillary muscles kissing to septum **(C)**.

We retrospectively analysed echocardiographic images diagnosed as DLVOTO with imaging obtained during Valsalva maneuver from 2008 to 2012 in a single tertiary referral hospital. DLVOTO was defined as a trans-LVOT peak PG higher than 30 mmHg at resting or provoked by a Valsalva maneuver, i.e., latent DLVOTO, in patients without fixed structural stenosis (Figure 1). Patients were excluded if they have classical HCM defined as asymmetric septal hypertrophy extend to mid ventricle not just confined to upper basal septum, septal to posterior wall ratio >1.5, acute myocardial infarction, stress induced cardiomyopathy, more than a moderate degree of valvular heart disease, unstable hemodynamics which might potentially induce transient-DLVOTO or poor echocardiographic images. Also, we randomly selected control group who had roughly similar age and gender distribution, but not had DLVOTO and cardiovascular disease, to study population in the same echo database and analysed their echocardiographic images. Study protocols were approved by our institutional review board, and informed consent from patients was waived due to the retrospective nature of the study.

### Conventional echocardiography with Valsalva maneuver

Each patient underwent a complete standard transthoracic echocardiography. The LV dimensions and septal and posterior wall thickness were measured at end-diastole and end-systole in the two-dimensional parasternal long axis or short axis views. LV ejection fraction was calculated using the modified Quinones’ method [7]. LV mass was measured by Devereux’s methods as recommended by the American Society of Echocardiography [7]. Relative wall thickness was calculated as twice the posterior wall thickness at end-diastole divided by the LV end-diastolic dimension. The left atrial volume was measured using the prolate ellipsoidal method at the point of LV end-systole when the left atrial size was maximal. From the apical window, a 1 mm pulsed Doppler sample volume was placed at the mitral valve tip, and mitral flow velocities from 5 to 10 cardiac cycles were recorded. Peak early (E) and late (A) mitral inflow velocities were also measured. Mitral annular velocity was measured by tissue Doppler imaging using the pulsed wave Doppler mode. The filter was set to exclude high frequency signal, and the Nyquist limit was adjusted to a range of 15 to 20 cm/s. Gain and sample volume were minimized to allow for a clear tissue signal with minimal background noise. Systolic (S’), early (e’) and late diastolic velocities of the mitral annulus were measured from the apical 4-chamber view with a 2- to 5-mm sample volume placed at the septal corner of the mitral annulus. Peak velocity of tricuspid regurgitation was measured and PASP was calculated as 4xTRV^2^ + RA pressure, where RA pressure was estimated according to IVC diameter and its respiratory variations [8]. In cases of systolic color flow acceleration in the LVOT, trans-LVOT peak PG was measured with continuous wave Doppler both at rest and during a Valsalva maneuver.

### End-systolic wall stress calculations

Before echocardiography, all the patients underwent blood pressure measurements in the upper arm with an automatic sphygmomanometer (TM-2665P, AND, CA). End-systolic systemic pressure was calculated as (2х systolic blood pressure + diastolic blood pressure)/3. The left ventricular end-systolic meridional wall stress was calculated by the method of Grossman et al. [9] Left ventricular wall stress (g/cm^2^) at the end of systole was calculated as (1.35)*(P_es_)*(D_es_)/(4 )*(h_es_)*[l + (h_es_///D_es_)], where P_es_ is the left ventricular pressure (mm Hg) at end-systole determined by the end-systolic blood pressure plus the end-systolic peak PG across the LVOT, D_es_ and h are the LV internal dimension and posterior wall thickness (cm) at end-systole, respectively, 1.35 is a conversion factor (mm Hg to g/cm), and 4 is a geometric factor that results from conversion of the radius to the internal dimension [10].

### Measurements of LV geometry associated with DLVOTO

**Figure 2:**
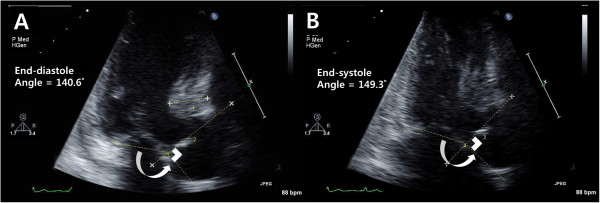
**Assessment of geometry-associated left ventricular outflow tract obstruction.** Measurements of basal septal wall thickness at end-diastole, the angle between the ascending aorta and the plane of the mitral valvular orifice at both end-diastole **(A)** and end-systole **(B)** from 3-chamber view images.

Interventricular basal septal thickness was measured at end-diastole in apical 3-chamber view images. The angle between the ascending aorta and the plane of the mitral valvular orifice was measured at both end-diastole and end-systole from 3-chamber views (Figure 2). Size of papillary muscles was measured in patients with prominent papillary muscle group in every imaging view. The presence of systolic anterior motion (SAM) of mitral valve and chordae was also reviewed from all the echo images.

### Statistical analysis

Clinical characteristics and echocardiographic parameters are presented as mean ± standard deviation for continuous variables and number (percentage) for categorical variables. Correlation analysis was performed between continuous variables using the Pearson correlation coefficient. Comparisons of echocardiographic findings between controls and study group were performed by independent t-test. Comparison of clinical and echocardiographic variables among subgroups was performed by ANOVA with post-hoc analysis via Bonferroni correction. The trans-LVOT-PG before and after a Valsalva maneuver was compared using the paired t-test. Variables with *p* < 0.10 in univariate analysis were included in the multivariable regression analysis. All the analyses were performed using SPSS (version 20.0, IBM, USA), and *p* values less than 0.05 were considered significant.

## Results

### Baseline clinical and echocardiographic characteristics

Among 33,060 primary transthoracic echo reports, total of 168 patients (0.5% of total primary cases) were studied after exclusion of cases with any exclusion criteria. Referral reasons for echocardiography were evaluations for chest pain or previous coronary artery disease in 27 (16%), ECG abnormalities in 15 (9%), dyspnea on exertion in 21 (13%), and evaluation for origin of cardiac murmur in 5 (3%), evaluation for target organ damage in hypertension or diabetes in 93 (55%) or evaluation for cause of syncope or dizziness in 7 (4%). Among them, 135 (80%) had latent DLVOTO. Mean age was 71 ± 11 years and 98 (58%) patients were women. One hundred thirty-six patients (81%) had a history of hypertension and 54 (32%) had diabetes. The mean LV end-diastolic dimension and ejection fraction were 39.3 ± 4.6 mm and 69.0 ± 5.7%, respectively. The patients almost had no regional wall motion abnormality except for few patients (n = 2). The body surface area corrected LV end-diastolic dimensional index (24.1 ± 3.1 mm/m^2^ vs. 26.7 ± 2.9 mm/m^2^, p < 0.001) and end-systolic dimensional index (15.3 ± 2.1 mm/m^2^ vs. 17.5 ± 2.5 mm/^2^, p < 0.001) were significantly lower than those of age- and sex- matched controls (n = 30). Relative wall thickness was significantly higher than controls (0.55 ± 0.10 vs. 0.46 ± 0.07, p < 0.001), suggesting significant concentric remodelling. Baseline clinical and echocardiographic variables are described in Table 1. The basal septal thickness (17.2 ± 2.57 mm vs. 13.0 ± 1.6 mm, p < 0.001) and the angle between the ascending aorta and mitral annular plane (152.4 ± 12.3° vs. 145.6 ± 11.4°, p = 0.006) were significantly higher compared to age- and sex-matched controls. The angle significantly increased from 152.4 ± 12.3° at end-diastole to 162.2 ± 11.8° at end-systole (p < 0.001). The trans-LVOT peak PG increased from 21.3 ± 19.8 mmHg to 46.9 ± 21.6 mmHg during a Valsalva maneuver. Basal septal thickness at end-diastole was not significantly different in hypertensive or diabetic patients compared to their counterparts. Basal septal thickness did not correlate with age, but the angle between the ascending aorta and mitral annular plane increased significantly with age (r = 0.179, p = 0.02), and this relationship remained significant after adjusting for hypertension, diabetes and sex (ß = 0.240, p = 0.003).

**Table 1:** Baseline clinical and echocardiographic characteristics according to the presence of latent obstruction

	**Control**	**Total**	**Resting DLVOTO**	**Latent DLVOTO**	^**§**^**P**
	**(n = 30)**	**(n = 168)**	**(n = 33)**	**(n = 135)**	
**Age**, yrs	70.5 ± 1.7	71.0 ± 10.9	68.5 ± 10.4	71.7 ± 11.0	0.134
**Women**, n (%)	17 (57)	98 (58)	19 (58)	79 (59)	0.922
**Hypertension**, n (%)	14 (47)	136 (81)	25 (76)	111 (82)	0.397
**Diabetes**, n (%)	5 (17)	54 (32)	7 (21)	47 (35)	0.134
**SBP**, mmHg	136.3 ± 14.6	129.0 ± 20.7*	122.9 ± 17.6	130.3 ± 21.2	0.095
**DBP**, mmHg	78.8 ± 7.5	71.2 ± 11.8†	71.4 ± 13.4	71.2 ± 11.5	0.948
**LVEDD**, mm	42.8 ± 3.4	39.3 ± 4.6†	39.1 ± 5.3	39.3 ± 4.4	0.785
**LVESD**, mm	28.1 ± 3.0	25.0 ± 3.3†	24.7 ± 3.8	25.0 ± 3.2	0.635
**LVEDD/BSA**, mm/m^2^	26.7 ± 2.9	24.1 ± 3.1†	23.6 ± 3.5	24.2 ± 3.0	0.303
**LVESD/BSA**, mm/m^2^	17.5 ± 2.5	15.3 ± 2.1†	14.9 ± 2.5	15.4 ± 2.0	0.255
**LV ejection fraction**, %	66.5 ± 6.1	69.0 ± 5.7*	69.5 ± 5.9	68.9 ± 5.6	0.558
**LV mass index**, g/m^2^	85.7 ± 15.7	89.0 ± 25.9	94.0 ± 31.2	87.7 ± 24.4	0.290
**BST**, mm	1.30 ± 0.16	1.72 ± 0.26†	1.76 ± 0.24	1.72 ± 0.26	0.372
**PWTd**, mm	9.67 ± 1.24	10.62 ± 1.70*	11.09 ± 1.76	10.51 ± 1.67	0.077
**PWTs**, mm	13.43 ± 1.72	14.37 ± 1.86*	14.82 ± 2.13	14.26 ± 1.78	0.123
**IVTd**, mm	10.07 ± 1.44	11.46 ± 1.96†	11.97 ± 2.20	11.34 ± 1.88	0.096
**IVTs**, mm	13.43 ± 1.72	15.02 ± 2.20†	15.33 ± 2.16	14.95 ± 2.21	0.369
**Relative wall thickness**	0.45 ± 0.07	0.55 ± 0.10†	0.58 ± 0.11	0.54 ± 0.10	0.084
**E/A ratio**	0.79 ± 0.13	0.69 ± 0.37	0.72 ± 0.32	0.68 ± 0.38	0.615
**E/e’**	10.1 ± 3.2	13.9 ± 5.1†	15.8 ± 7.1	13.6 ± 4.5	0.123
**S’**, cm/s	9.5 ± 1.3	8.4 ± 2.2	9.5 ± 3.3	8.1 ± 2.0	0.048
**LA volume index**, ml/m^2^	21.7 ± 5.3	25.6 ± 10.9*	27.5 ± 18.6	25.1 ± 8.0	0.480
**LVESWS**, g/cm^2^	57.1 ± 12.4	49.8 ± 13.0*	57.8 ± 16.9	48.0 ± 11.4	0.007
**Any diuretics**, n (%)	4 (13)	56 (33)*	12 (36)	44 (33)	0.680
**CCB- dihydropyridine**, n (%)	2 (7)	80 (48)†	17 (52)	63 (47)	0.398
**CCBs-non-dihydropyrine**, n (%)	0 (0)	7 (4)†	0 (0)	7 (4)	
**Any BBs**, n (%)	2 (7)	44 (26)*	7 (21)	37 (27)	0.468
**Angle at end-diastole**, °	145.6 ± 11.4	152.4 ± 12.3*	151.8 ± 11.9	152.5 ± 12.5	0.767
**Angle at end-systole**, °	154.8 ± 11.6	162.2 ± 11.8*	162.1 ± 11.2	162.3 ± 12.0	0.933
**Δ PG**, mmHg		29.5 ± 17.5	29.1 ± 29.1	29.6 ± 15.3	0.946
**PASP**, mmHg	24.1 ± 5.3	28.8 ± 7.9*	32.2 ± 8.3	28.0 ± 7.6	0.007

*p < 0.05, †p < 0.01, ^§^resting DLVOTO vs. latent DLVOTO, SBP, systolic blood pressure; DBP, diastolic blood pressure; LV, left ventricular; LVEDD, left ventricular end-diastolic dimension; LVESD, left ventricular end-systolic dimension; BSA, body surface area; BST, basal septal thickness; PWTd, end-diastolic left ventricular posterior wall thickness; LA, left atrial; LVESWS, left ventricular end-systolic wall stress; CCB, calcium channel blocker; BB, beta blocker; PG, pressure gradient.

### Resting and latent DLVOTO

Patients with resting DLVOTO had higher LVESWS (p = 0.007), lower S’ (p = 0.048) and a higher pulmonary arterial systolic pressure (p = 0.007) than those with latent DLVOTO. However, the prevalence of concomitant hypertension, diabetes and anti-hypertensive medications were not different. In the latent DLVOTO group, the indexed LV end-diastolic dimension was inversely and significantly correlated with an increase of the PG between the resting state and the Valsalva maneuver (r = −0.224, p = 0.01). However, other parameters such as angle and basal septal thickness were not significantly correlated with peak PG change.

### Morphologic classification related to DLVOTO

**Figure 3:**
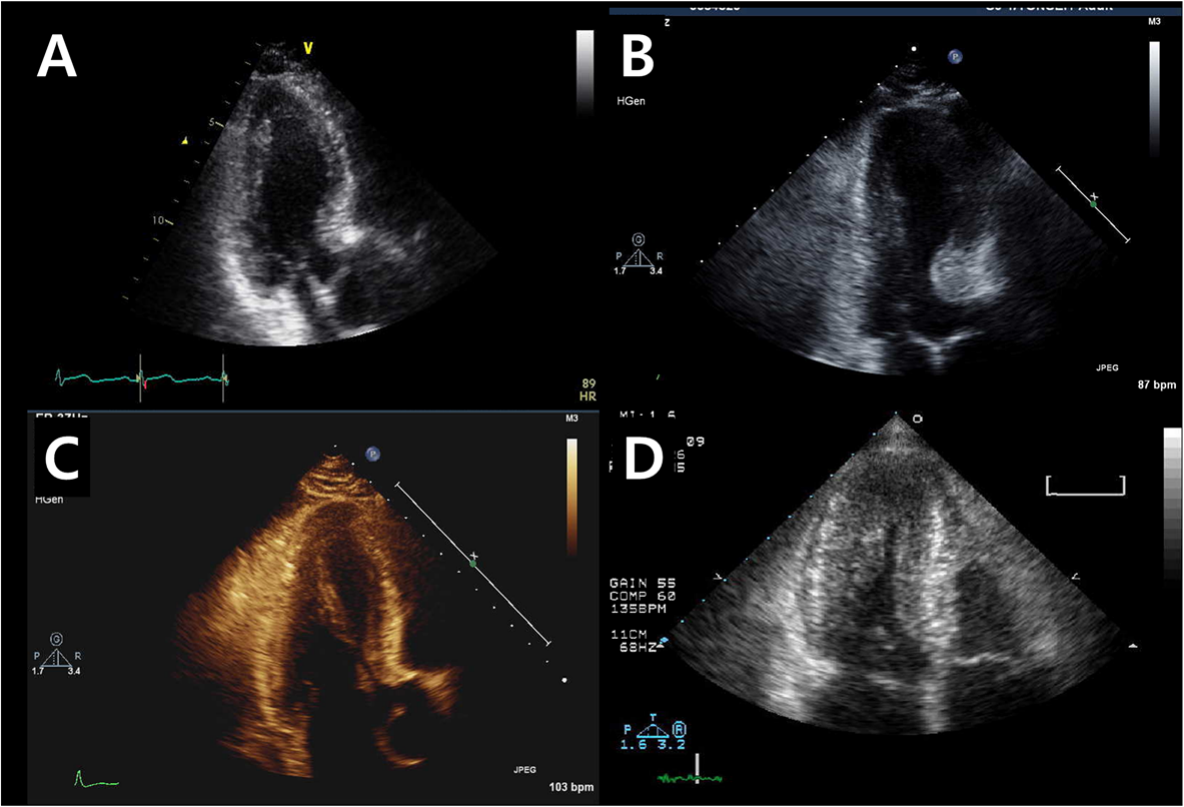
**Representative case for each subgroup.** Pure sigmoid septum **(A)**, sigmoid septum with basal septal hypertrophy **(B)**, prominent papillary muscle **(C)** and small LV cavity due to concentric remodelling **(D)**.

Study patients were classified as either having a pure sigmoid septum, defined as basal septal bulging but diastolic thickness ≤ 15 mm (n = 14); a sigmoid septum with basal septal hypertrophy higher than 15 mm (n = 85); a prominent papillary muscle (PM) (n = 20), defined as a visually large PM which occlude the LV cavity during systole or >1/2 LV end-systolic dimension and maximal thickness of PM in short axis view higher than 11.2 mm or longer than 2.6 cm in 2, 3 or 4 chamber view; or a small LV cavity with concentric remodelling or hypertrophy (n = 49) (Figure 3). The prominent PM group was younger, had a higher S’ and a lower E/e’ than other groups. The frequency of beta-blocker usage was lower in the prominent PM group. However, resting and Valsalva-induced trans-LVOT peak PGs were not different among subgroups (Table 2). Leaflets SAM was observed in 31 patients (19%), chordal SAM in 80 (48%), papillary muscle kissing to hypertrophied septum in 38 (23%), mixed type in 3 (2%) and non-above category was in 16 (10%). Patients with leaflets SAM had higher restign trans-LVOT PG. Analyses of their characteristics are described in the Table 3.

**Table 2:** Comparisons of clinical and echocardiographic parameters among morphologically classified subgroups

	**Pure sigmoid septum**	**Sigmoid septum with BSH**	**Prominent PM**	**Concentric remodelling**	**P**
	**(n = 14)**	**(n = 85)**	**(n = 20)**	**(n = 49)**	
**Age**, years	75.4 ± 9.5	72.3 ± 9.7	65.6 ± 9.7	69.9 ± 12.9	0.032
**Women**, n (%)	11 (79)	51 (60)	10 (50)	26 (53)	0.310
**Hypertension**, n (%)	14 (100)	70 (82)	16 (80)	36 (74)	0.158
**Diabetes**, n (%)	8 (57)	24 (28)	6 (30)	16 (33)	0.199
**SBP**, mmHg	124.9 ± 15.0	131.2 ± 21.0	125.3 ± 17.5	127.8 ± 22.7	0.550
**DBP**, mmHg	69.8 ± 10.2	72.3 ± 11.6	68.1 ± 12.9	71.0 ± 12.1	0.546
**LVEDD**, mm	38.7 ± 3.5	39.6 ± 4.2	39.7 ± 4.1	38.8 ± 5.7	0.742
**LVESD**, mm	25.4 ± 2.5	25.1 ± 3.0	25.8 ± 4.2	24.4 ± 3.6	0.361
**LVEDD/BSA**, mm/m^2^	24.9 ± 3.0	24.7 ± 3.1	22.9 ± 2.3	23.5 ± 3.1	0.028
**LVESD/BSA**, mm/m^2^	16.3 ± 1.8	15.6 ± 2.1	14.8 ± 2.0	14.7 ± 2.1	0.023
**LV ejection fraction**, %	66.9 ± 4.5	69.2 ± 5.7	67.9 ± 6.7	69.7 ± 5.4	0.298
**Angle at end-systole**, °	166.4 ± 14.6	161.0 ± 12.1	164.2 ± 10.7	162.4 ± 10.7	0.356
**Angle at end-diastole**, °	156.7 ± 13.4	151.3 ± 12.1	155.4 ± 12.5	151.7 ± 12.3	0.300
**BST**, mm	13.9 ± 1.1	18.3 ± 2.1	14.9 ± 1.4	17.4 ± 2.5	<0.001
**LVMI**, g/m^2^	74.4 ± 16.7	92.1 ± 25.3	77.2 ± 13.8	92.6 ± 30.2	0.012
**LA volume index**, ml/m^2^	27.3 ± 8.3	27.0 ± 13.0	21.9 ± 5.9	24.1 ± 8.4	0.176
**E/e’**	14.6 ± 5.4	14.3 ± 4.9	10.9 ± 3.3	14.5 ± 5.6	0.042
**S’**, cm/s	8.1 ± 2.0	8.3 ± 2.0	10.0 ± 3.1	7.8 ± 2.0	0.004
**Latent DLVOTO**, n (%)	14 (100)	16 (78)	17 (85)	38 (78)	0.227
**LVOT PG**_**resting**_, mmHg	14.8 ± 7.0	23.2 ± 23.1	17.5 ± 13.1	21.6 ± 18.0	0.393
**LVOT PG**_**Valsalva**_, mmHg	43.2 ± 11.8	47.4 ± 25.1	46.2 ± 13.7	47.4 ± 20.4	0.919
**ΔPG**, mmHg	28.4 ± 16.3	29.5 ± 18.8	30.6 ± 12.3	29.4 ± 18.0	0.988
**LVESWS**, g/cm	53.9 ± 10.4	51.9 ± 14.1	51.1 ± 14.3	44.5 ± 9.8	0.011

DLVOTO, dynamic left ventricular outflow tract obstruction; See abbreviations in Table 1.

**Table 3:** Classification according to presence or patterns of systolic anterior motion of mitral valve

	**Leaflet SAM**	**Chordal SAM**	**PM kissing**	**Mixed**	**None of above**	**P**
	**(n = 31)**	**(n = 80)**	**(n = 38)**	**(n = 3)**	**(n = 16)**	
Age, years	71.4 ± 11.0	72.2 ± 9.6	67.2 ± 13.5	69.3 ± 17.8	74.1 ± 7.5	0.140
Women, n (%)	18 (13)	27 (34)	14 (37)	2 (67)	9 (56)	0.091
Hypertension, n (%)	20 (65)	67 (84)	34 (90)	2 (67)	13 (81)	0.091
SBP, mmHg	123.9 ± 17.2	130.8 ± 19.4	131.6 ± 23.6	120.0 ± 5.7	123.7 ± 26.5	0.409
DBP, mmHg	71.8 ± 11.3	71.4 ± 11.3	72.8 ± 13.4	71.0 ± 8.5	64.7 ± 10.3	0.320
LVEDD/BSA, mm/m^2^	23.8 ± 2.7	24.4 ± 2.7	23.6 ± 3.2	28.0 ± 9.8	24.3 ± 4.3	0.255
LVESD/BSA, mm/m^2^	15.0 ± 2.3	15.6 ± 2.0	14.9 ± 2.1	17.2 ± 4.3	15.2 ± 2.4	0.271
LV ejection fraction, %	70.3 ± 5.1	68.1 ± 5.8	69.5 ± 5.7	71.0 ± 7.1	69.6 ± 5.9	0.355
Angle at end-systole, °	160.3 ± 9.9	161.2 ± 11.9	165.5 ± 12.4	162.2 ± 11.4	163.5 ± 13.0	0.338
Angle at end-diastole, °	151.3 ± 10.0	151.1 ± 12.8	154.8 ± 13.2	150.6 ± 10.1	155.0 ± 12.1	0.505
BST, mm	15.7 ± 2.5	15.6 ± 2.7	14.1 ± 2.0	15.9 ± 3.3	15.7 ± 1.7	0.024
LVMI, g/m^2^	96.4 ± 30.8	87.5 ± 20.4	87.1 ± 24.1	86.4 ± 30.2	86.7 ± 40.9	0.540
LA volume index, ml/m^2^	27.6 ± 18.8	24.2 ± 7.2	27.0 ± 7.8	34.1 ± 17.6	24.0 ± 10.9	0.340
E/e’	15.0 ± 5.5	14.0 ± 5.2	13.4 ± 4.4	13.1 ± 6.1	12.6 ± 0.6	0.716
S’, cm/s	8.5 ± 2.3	8.1 ± 1.8	8.4 ± 2.6	6.0 ± 1.4	9.7 ± 3.1	0.076
Latent DLVOTO, n (%)	16 (52)	74 (93)	32 (84)	3 (100)	10 (63)	<0.001
LVOT PG_resting_, mmHg	36.7 ± 28.8	15.6 ± 15.0	18.3 ± 14.3	12.6 ± 6.3	27.1 ± 15.8	<0.001
LVOT PG_Valsalva_, mmHg	64.3 ± 39.9	43.4 ± 13.9	42.7 ± 13.0	41.4 ± 20.4	45.3 ± 11.3	<0.001
LVESWS, g/cm	52.1 ± 15.5	49.8 ± 13.1	48.7 ± 10.6	38.7 ± 4.8	49.2 ± 14.0	0.633

SAM, systolic anterior motion of mitral valve. See abbreviations in the Tables 1 and 2.

### Clinical and hemodynamic impact of DLVOTO

In all groups, a higher peak trans-LVOT PG was correlated to higher E/e’ (r = 0.246, p = 0.002), pulmonary arterial systolic pressure (PASP, r = 0.317, p < 0.001), systolic blood pressure (r = −0.142, p = 0.081) and relative wall thickness (r = 0.141, p = 0.070). In multivariate analysis, resting trans-LVOT PG correlated to PASP (ß = 0.226, p = 0.019) after adjustment for systolic blood pressure, relative wall thickness, and E/e’.

## Discussion

In this study, we found that, even in patients without overt HCM, DLVOTO, especially the latent form, is not infrequently seen in the absence of acute volume depletion or hemodynamic instability. This group of patients mainly has basal septal bulging and concentric remodelling that is classically assumed to be an aging-related change [11,12]. However, diverse morphological subgroups also exist. A significant portion of patients have leaflets or chordal SAM. Of those subgroups, patients with prominent PMs had distinct clinical and LV functional characteristics suggesting a different etiology. In this group of patients, a higher trans-LVOT PG results in a higher pulmonary arterial pressure through diastolic dysfunction. Thus, DLVOTO-relieving medication may potentially reduce pulmonary pressure in this group of patients.

### Diverse geometric changes related to DLVOTO

This group of patients had a small LV cavity and increased basal septal thickness with a more angulated ascending aorta compared to age- and sex-matched controls. Classically, basal septal bulging due to focally increased wall thickness or increased septal to ascending aorta angle [11] with concentric LV remodelling [12] has been accepted as part of the aging process. Although the high prevalence of hypertension in the enrolled patients is possibly due to referral bias, basal septal thickness and aortic-angle were not different between hypertensive patients and their counterparts, which suggests that hypertension is not the main cause of DLVOTO related changes of LV geometry. Gender predominance was not observed. Our study also supports the finding that age is significantly correlated with the degree of aortic-angulation. This change is usually termed either sigmoid septum or basal septal hypertrophy, resulting in some confusion. Some previous reports have described basal septal hypertrophy as a subtype of HCM [13]. According to the results of this study, basal septal hypertrophy does not result in a difference in clinical characteristics compared to a purely sigmoid septal outflow tract obstruction. This is in contrast to the differences seen in the prominent PM group, suggesting that basal septal hypertrophy is more likely an aging-related change rather than a subtype of HCM. The current study also supports previous studies which showed that hypertrophied PMs were related to DLVOTO in the absence of LV hypertrophy [14]. To prevent misdiagnosis, when DLVOTO is detected, both an age-related sigmoid septum and PM hypertrophy should be considered. Thorough evaluation for the possibility of HCM might be needed in this subgroup. This distinction also suggests that basal septal hypertrophy is less likely to be a subtype of HCM. Another interesting finding is lots of patients had SAM or chordal SAM in this population, which is consistent with previous observation [15]. Therfore LV geometric change in combination with abnormal mitral apparatus abnormalities such as systolic movement of mitral leaflets, chordae and papillary muscle may contribute developments of DLVOTO [16,17].

### Clinical and hemodynamic characteristics of DLVOTO

Elderly people frequently complain of dyspnea on exertion due to diastolic dysfunction and reactive pulmonary venous hypertension. Therefore, aging is thought to be the leading cause of heart failure with a preserved ejection fraction [18]. The usual treatment for this group of patients is diuretics to relieve symptoms. However, not all these patients respond well to diuretics, and some patients suffer from significant orthostatic hypotension [19]. In this regard, the patients enrolled in this study share similar risk factors for the development of heart failure with preserved ejection such as aging, a higher prevalence of hypertension, and diastolic dysfunction with concentric remodelling [20]. Although LV ejection fraction was significantly higher in DLVOTO group compared to control, trans-LVOT- PG was not significantly correlated to LV ejection fraction. Therefore, geometrical change around LVOT might be stronger determinants than LV ejection fraction for degree of DLVOTO. According to this study, a higher trans-LVOT PG was associated with a higher pulmonary arterial pressure, so particular attention should be paid when selecting medications in this group of patients. In this regard, beta blockers or non-dihydropyridine-based calcium channel blockers, rather than diuretics, can improve symptoms and outcomes. This group of patients would be a potential subgroup of heart failure with preserved ejection fraction. Therefore, their managements should be individualized.

### Study limitations

This study has several limitations. First, due to the nature of this retrospective study performed in a tertiary referral hospital, there may be some referral bias, and the actual prevalence of concomitant risk factors could not be truly measured. Additionally, Valsalva maneuver was not routinely done in all patients but only in patients with color flow turbulence or acceleration at rest in our echo-lab, the actual prevalence of latent DLVOTO might be underestimated. Secondly, although patients with secondary DLVOTO related to HCM or other conditions were excluded, complete evaluation by invasive hemodynamic status was not performed. Thirdly, classification based on basal septum thickness, LV concentric remodelling and visually assessed PM morphology is somewhat arbitrary. However, individual subtypes have been reported in previous studies [5,11,14], so the application of a novel classification system to this study group seems appropriate.

## Conclusions

DLVOTO can develop due to various geometric changes, but patients with a prominent PM have distinct characteristics suggestive of atypical HCM rather than age-related changes. A higher trans-LVOT PG results in a higher pulmonary arterial pressure through diastolic dysfunction. Therefore, LVOTO-relieving medication might reduce the occurrence or severity of symptoms by reducing pulmonary pressure in this group of patients.

## Competing interests

The authors declare that they have no competing interest.

## Authors’ contributions

EYC and JJC made the study design. JJC, HC and YWY analysed echocardiography images. EYC, JJC and HC collected the echocardiographic and clinical data. EYC and JJC analysed and interpreted the data. HC made a proposal and did IRB processing job. JJC, HC, YWY and EYC wrote manuscript. JHY, YWY, JYK, BKL, PKM, BKH, SJR and HMK collected the clinical data and gave comments on the manuscript. All authors read and approved the final manuscript.
